# Anatomy of provincial level inequality in maternal mortality in China during 2004–2016: a new decomposition analysis

**DOI:** 10.1186/s12889-020-08830-2

**Published:** 2020-05-24

**Authors:** Xinyu Zhang, Yingfeng Ye, Chaowei Fu, Guanshen Dou, Xiaohua Ying, Mengcen Qian, Shenglan Tang

**Affiliations:** 1 Department of Health Economics, School of Public Health, Fudan University, Shanghai, China; 2 Department of Social Medicine, School of Public Health, Fudan University, Shanghai, China; 3 NHC Key Laboratory of Health Technology Assessment (Fudan University), Shanghai, China; 4 Duke Global Health Institute, Duke University, Durham, USA

**Keywords:** Maternal health, Health inequality, Decomposition analysis, China

## Abstract

**Background:**

The maternal mortality ratio (MMR) is an important indicator of maternal health and socioeconomic development. Although China has experienced a large decline in MMR, substantial disparities across regions are still apparent. This study aims to explore causes of socioeconomic related inequality in MMR at the province-level in China from 2004 to 2016.

**Methods:**

We collected data from various issues of the China Health Statistics Yearbook, China Statistics Yearbook, and China Population and Employment Statistics Yearbook to construct a longitudinal sample of all provinces in China. We first examined determinants of the MMR using province fixed-effect models, accounted for socioeconomic condition, health resource allocation, and access to health care. We then used the concentration index (CI) to measure MMR inequality and employed the direct decomposition method to estimate the marginal impact of the determinants on the inequality index. Importance of the determinants were compared based on logworth values.

**Results:**

During our study period, economically more deprived provinces experienced higher MMR than better-off ones. There was no evidence of improved socioeconomic related inequality in MMR. Illiteracy proportion was positively associated with the MMR (*p* < 0.01). In contrast, prenatal check-up rate (*p* = 0.05), hospital delivery rate (*p* < 0.01) and rate of delivery attended by professionals (*p* = 0.02) were negatively associated with the MMR. We also find that higher maternal health profile creation rate (*p* < 0.01) was associated with a pro-poor change of MMR inequality.

**Conclusion:**

Access to healthcare was the most important factor in explaining the persistent MMR inequality in China, followed by socioeconomic condition. We do not find evidence that health resource allocation was a contributing factor.

## Background

The maternal mortality ratio (MMR), defined as the number of maternal deaths per 100,000 live births, is an important indicator of maternal health as well as socioeconomic development in a country or region [1]. Due to the significant relevance of this indicator, the United Nations has set specific targets to improve maternal health based on it. For example, the Millennium Development Goals aimed to reduce the MMR by three quarters during 1990 to 2015, with a special focus on low- and middle- income countries. Subsequently, the Sustainable Development Goals (SDGs) further extends the target to a global MMR of less than 70 per 100,000 live births and the achievement of a 68% reduction by 2030.

China, with its rapid economic development during the past 30 years, has successfully reduced its MMR. In 1991, the ratio was 80.0 per 100,000 live births; in 2016, it dropped to 19.9 per 100,000 live births [2]. The change corresponded to an annual decrease of 5.18%. The country, as a whole, has achieved the SDG target before the suggested deadline by the United Nations. However, a close inspection of the ratio at provincial level suggests substantial and persistent regional disparities despite overall reduction [3–5]. For example, in 2016, Tibet (a less-developed province in western China) has an MMR level (109.9/100,000) that is 5.5 times of the national average and 50 times of the Jiangsu province (a well-developed province in eastern China) (2.2/100,000). These numbers suggest persistent disparities associated with socioeconomic conditions, which has posed great challenges to the central and local governments of China in pursuing health equality [6, 7].

Indeed, over the past decades, China has made tremendous efforts to improve the equity in maternal and child health. Several rounds of the “Reducing Maternal Mortality and Eliminating Neonatal Tetanus” programs were initiated in 2000, 2002 and 2005 to promote prenatal care utilization and hospital delivery with a focus on rural provinces [5]. Since 2003, hospital delivery has been covered by the New Cooperative Medical Scheme, which is a social health insurance program for almost the entire Chinese rural population. In 2009, the hospital delivery subsidy program in rural area was carried out [8]. In the same year, the equalization of basic public health services policy was adopted, in which maternal health management was emphasized.

Despite the great policy interests, root causes of regional disparities in MMR in China are less understood. Using aggregate-level data, previous studies have identified that GDP per capita, female illiteracy rate, density of licensed doctors, and hospital delivery are important determinants of MMR [1, 3, 7]. However, contributing factors of health inequality and those of the underlying health outcomes are not necessarily identical [9–11]. Other studies on maternal and child health in China investigated reasons for inequality of healthcare utilization [12–15] and child health outcomes [11, 16]. We found no studies that examine the causes of inequality of MMR in China.

In this study, we utilized a new decomposition technique to explore causes of socioeconomic related inequality in MMR at the provincial level in China from 2004 to 2016. From various issues of the China Health Statistics Yearbook, we constructed a longitudinal sample of all 31 provinces with extensive characteristics from three broad aspects including socioeconomic condition, health resources allocation, and access to maternal health care. The three aspects were developed under the framework proposed by Countdown to 2015’s Health Systems and Policies Working Group [7]. The inequality level was measured by concentration index (CI). We first examined the determinants of MMR using province fixed-effect models. We then followed the technique developed by Kessels and Erreygers [17] (the direct decomposition method, onwards) to estimate the marginal impact of the aforementioned determinants on the inequality index. The relative importance of the factors was compared based on logworth values. To facilitate comparison with the literature [18, 19], we also presented results from the decomposition method proposed by Wagstaff et al. [20] (WDW, onwards).

The empirical challenge to decompose the concentration index lies in its bivariate rank-dependent nature. The index relates an observation’s health levels to its relative rank of socioeconomic status. In the health inequality literature, the dominant procedure to decompose the bivariate rank-dependent index has been the WDW method [18, 19]. The WDW approach produces descriptive percentage-wise contributions of different factors to the observed health inequality level based on a regression analysis of the health outcome. However, the method has been largely criticized for its “one-dimensional” feature that may lead to biased conclusions because it focuses solely on the health variable and ignores the association between the factors and the socioeconomic rank [21, 22].

In contrast, the recently formulated approach, the direct decomposition method [17], recognizes the bivariate nature of rank dependent indicators. Consequently, it has been considered superior to the WDW decomposition approach. The outcome of the regression analysis in the direct regression approach is individual components of the inequality indicator, that is, the performance of an observation jointly for health and socioeconomic rank relative to the average of both dimensions. Results can be straightforwardly interpreted as marginal effects of the regression explanatory variables on the socioeconomic related inequality index, which is another important strength compared to the traditional WDW decomposition method.

## Method

### Data and variables

We constructed a longitudinal sample of all 31 provinces in mainland China for an extended time period from 2004 to 2016 by collecting data from various issues of the China Health Statistics Yearbook, China Statistics Yearbook and China Population, and Employment Statistics Yearbook. We obtained maternal mortality ratio (per 100,000 live births), gross domestic product (GDP) per capita, and an extensive set of variables by province and year: (1) for *socioeconomic condition*, we considered average annual household consumption (average total annual household expenditures on final consumption of goods and services), proportion of illiteracy, and proportion of college equivalent-educated persons; (2) for *health resource allocation*, we included public budget in health sector per capita, density of health providers in specialized maternal and child health hospitals (total number per 10,000 population), density of specialized maternal and child health hospitals (total number per 1,000,000 population), and bed size in maternal and child health hospitals per 100,000 population; (3) for *access to health care*, we considered maternity health insurance coverage (the proportion of urban employed population under social health insurance who opt in the maternity health insurance), premarital check-up rate, maternal health profiles creation rate (the number of health profiles created by maternal care providers over live births), coverage of maternal systematic management (the number of women over live births, who received early pregnancy test, at least five prenatal check-ups, delivery attended by professionals and postpartum visits up to 42 days after giving birth), prenatal check-up rate, postpartum visit rate, hospital delivery rate, and delivery attended by professionals rate. Table S1 summarizes the definition of variables used in the analysis.

### Measures for inequality

We used the standard concentration index to measure the relative socioeconomic inequality of maternal mortality ratio. We ranked each observation by GDP per capita and obtained a fractional rank for each observation in terms of this variable. The CI is defined as twice the covariance of the health variable and the fractional rank in terms of socioeconomic status over the mean of the health variable, which can then be written as

1 $$ CI=\frac{1}{\mu_{MM R}}2\mathit{\operatorname{cov}}\left( MM{R}_i,{F}_{GDP}\right)=\frac{1}{N}\sum \left(\tilde{f}_{i}\frac{MM{R}_i}{\mu_{MM R}}-\frac{MM{R}_i}{\mu_{MM R}}\right) $$

where *μ*_*MMR*_ is the mean of MMR, *F*_*GDP*_ is the fractional rank for each observation in terms of GDP per capita, and $\tilde{f}_{i}$ is the relative fractional rank. We obtained the concentration index using the Stata command “conindex.”

### Regression analysis of MMR

We first explored the determinants of the maternal mortality ratio using a fixed-effect model. For each province *i* in year *t*, we considered

2 $$ {MMR}_{it}=\beta {X}_{it}+{\tau}_t+{\delta}_i+{\varepsilon}_{it} $$

where *X*_*it*_ is a set of provincial characteristics as listed in Table 1, which includes socioeconomic condition, health resource allocation, and access to health care. *τ*_*t*_ is year fixed effects, which capture general impacts of the macro environment in each year. *δ*_*i*_ is province fixed effects, which capture time-invariant province-level characteristics that may affect the maternal mortality ratio. *ε*_*it*_ is the error term. To capture area specific time trends, we also controlled for a linear year trend for each individual province. The parameters of interest, *β*, is identified by variation in provincial characteristics for a given province over years. Standard errors were clustered at the provincial level.

**Table 1:** Sample means and sources of variation in province characteristics

	Mean	Standard deviation		
		Overall	Between-province	Within-province
	(1)	(2)	(3)	(4)
Maternal mortality ratio (per 100,000 live births)	29.83	(38.37)	(34.22)	(18.33)
GDP per capita (RMB)	34,086.08	(22,754.69)	(17,085.12)	(15,316.27)
Socioeconomic condition				
Average annual household consumption (RMB)	11,918.68	(8083.85)	(5931.95)	(5586.68)
Proportion of illiteracy (%)	7.90	(6.62)	(6.36)	(2.14)
Proportion of college educated (%)	9.99	(6.50)	(5.85)	(3.01)
Health resource allocation				
Public budget in health sector per capita (RMB)	495.19	(375.44)	(187.95)	(326.62)
Density of health providers in specialized maternal and child health hospitals (per 10,000 population)	1.57	(0.60)	(0.46)	(0.40)
Density of specialized maternal and child health hospitals (per 1,000,000 population)	3.05	(3.03)	(3.06)	(0.29)
Density of bedsize in maternal and child health hospitals (per 100,000 population)	10.14	(5.08)	(3.37)	(3.85)
Access to health care				
Maternity health insurance coverage (%)	49.32	(14.73)	(9.81)	(11.11)
Premarital check-up (%)	27.46	(30.53)	(17.00)	(25.53)
Maternal health profiles creation rate (%)	91.67	(8.53)	(6.76)	(5.32)
Coverage of maternal systematic management (%)	82.29	(14.34)	(11.50)	(8.79)
Prenatal check-up (%)	92.51	(7.19)	(5.79)	(4.37)
Postpartum visit rate (%)	89.26	(9.56)	(7.89)	(5.57)
Hospital delivery rate (%)	93.93	(11.34)	(8.03)	(8.14)
Delivery attended by professionals rate (%)	98.47	(4.07)	(3.18)	(2.59)
Observations	403			

Notes: Data source is various issues of China Health Statistics Yearbook, China Statistics Yearbook and China Population, and Employment Statistics Yearbook. The sample consists of all 31 provinces in mainland China over 13 years from 2004 to 2016. Sample means, overall standard deviations, between- and within-province deviations (in parentheses) are reported. RMB refers to Renminbi, which is the monetary unit in China

### Decomposition analysis

We then used a regression-based approach to decompose the causes of MMR inequality. Considering the “two-dimensional” feature of the concentration index, we adopted the direct regression method. Following Kessels and Erreygers [17], we calculated an individual component of the inequality index, which is given by

3 $$ {u}_i=\tilde{f}_{i}\frac{MM{R}_i}{\mu_{MM R}}-1 $$

where $\tilde{f}_{i}$ is the relative fractional rank for each observation in terms of GDP per capita. The *u*_*i*_ captures the deviation of the joint outcome with respect to GDP per capita and MMR from a reference position, where both relative fractional rank and relative MMR are at their average levels. To estimate marginal effects of province characteristics on the individual component of the concentration index, we replaced the outcome variable in eq. (2) with *u*_*i*_. The regression is then given by

4 $$ {u}_{it}=\gamma {X}_{it}+{\tau}_t+{\delta}_i+{\eta}_{it} $$

where *η*_*it*_ is the unexplained error term. Under the assumption that the expectation of the error term conditional on other control variables equals zero, *γ* can also be interpreted as the marginal effect of the control variable on the concentration index. To assess the importance of the explanatory variables in the above regression, we obtained logworth statistic values for each control variable in eq. (4). The logworth statistic is defined as –log_10_ (*p* value of the F test) [17].

We also decomposed the causes of MMR inequality using the conventional WDW approach. Based on the estimated coefficient *β* from regression eq. (2), we calculated the contribution percentage of each explanatory variable to the concentration index using the following equation

5 $$ concentration\%=\left(\frac{\beta \overline{x}}{\mu_{MMR}}\right)\frac{C{I}_k}{CI} $$

where $\overline{x}$ is the average of the explanatory variable, and *CI*_*k*_ is the concentration index of the explanatory variable. Similar to van Doorslaer et al. [23] and Sortsø et al. [24], we apply a “bootstrap” procedure to obtain standard errors for the estimated concentration percentage. We replicated the entire calculation using a random sample of the size of the original sample with replacement and repeated the whole process 500 times. Results from the 500 replicates were used to compute the standard errors of the calculated concentration percentage.

All the statistical analyses were performed using Stata 16.0 for Windows. We used 5% as the significance level and reported findings with a *p*-value less than 0.1 as marginally significant.

## Results

### Sample characteristics

The final sample consists of all 31 provinces in mainland China over 13 years from 2004 to 2016. Table 1 presents sample means and sources of variations of the variables included in the analysis. The range of MMR was between 1.2 and 310.4 per 100,000 live births. Around 89% of the variations in MMR came from heterogeneity across provinces. Although between-province variation was generally larger for most variables, we observed sufficient changes across time in a given province, which we exploited for the identification of the marginal effects of these variables on the MMR and individual components of the inequality indicator.

### Socioeconomic inequality in MMR

**Fig. 1:**
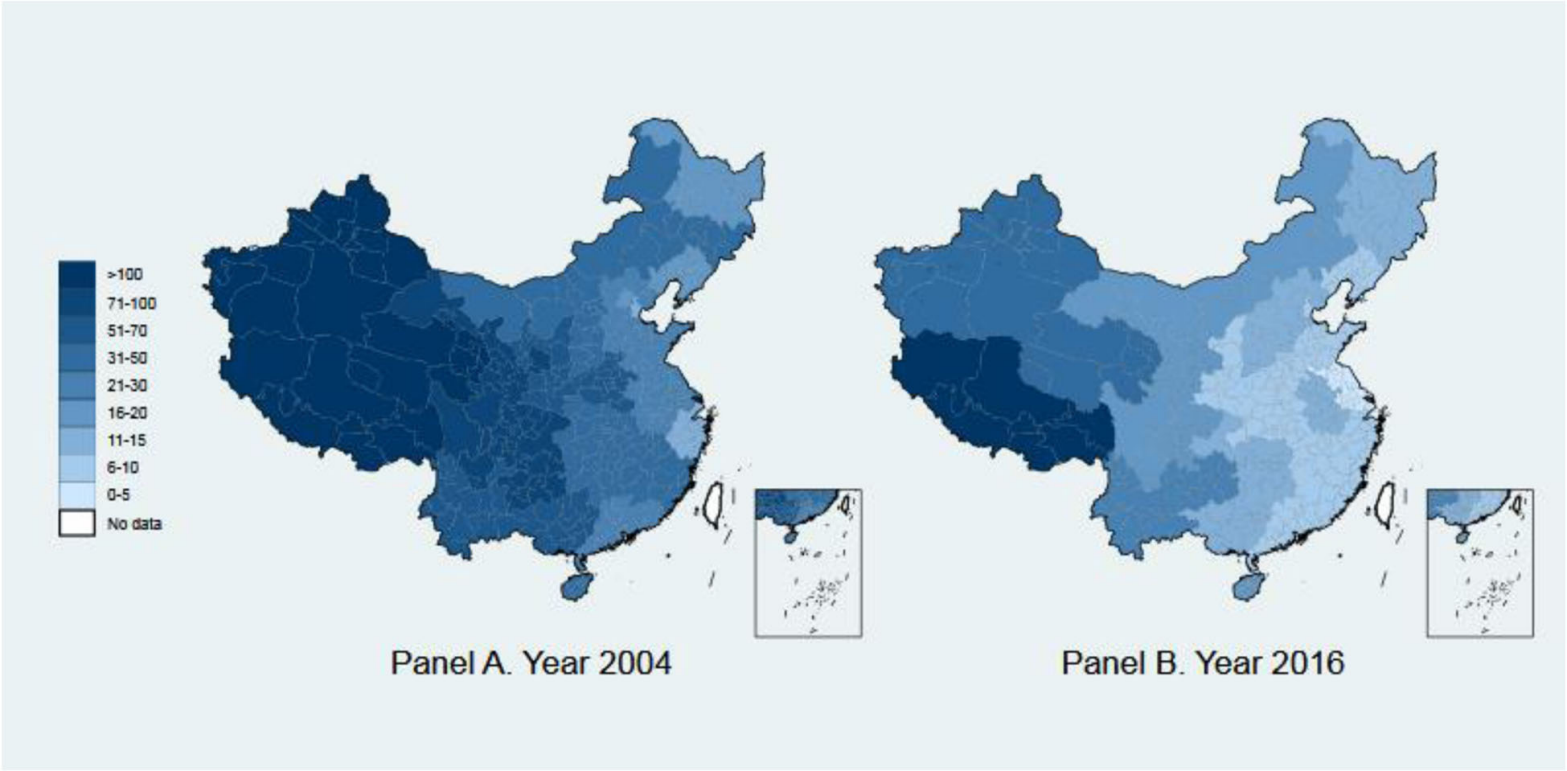
Maternal mortality ratio across provinces in China in 2004 and 2016, produced using Stata 16 for Windows. Notes: Data source is various issues of China Health Statistics Yearbook. The years 2004 and 2016 are the earliest and latest years with available maternal mortality ratio at province level

Panel A and B of Fig. 1 map MMR levels across provinces in 2004 and 2016, respectively. Although MMR decreased in almost all provinces, apparent distinctions between more developed eastern and less developed western areas can still be observed across years, suggesting that such regional disparities have been associated with socioeconomic levels.

**Fig. 2:**
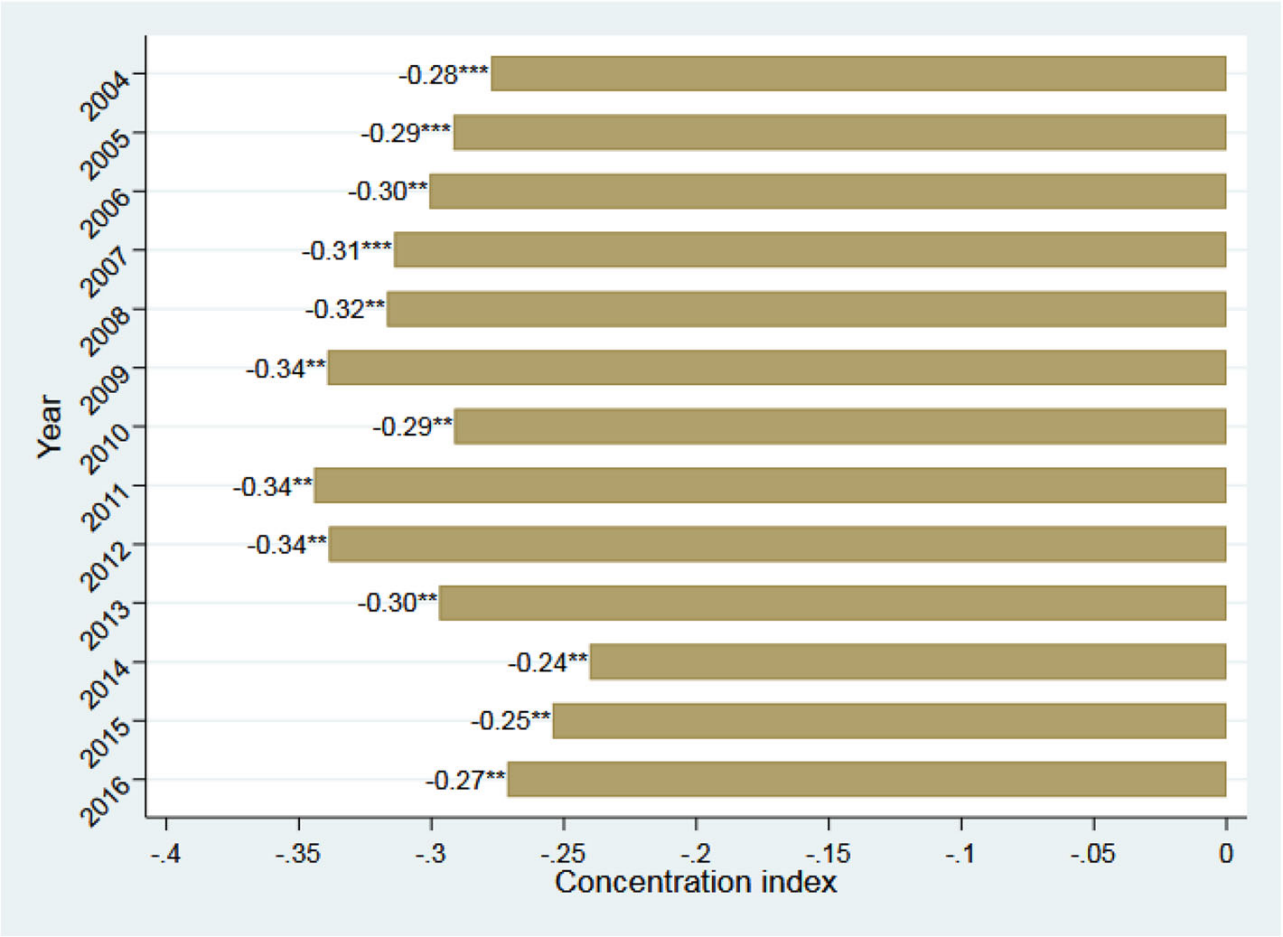
Concentration index of maternal mortality ratio by year, 2004–2016. Notes: Data source is various issues of China Health Statistics Yearbook. Concentration indices of maternal mortality ratio using fractional rank generated based on GDP per capita are presented in the graph

The concentration index for province maternal mortality ratio was − 0.367 (*p* < 0.001), indicating that higher maternal mortality ratio was more concentrated amongst poor provinces with low level of development. We find statistically significant evidence for relative socioeconomic inequality. Figure 2 presents the index by each year. We find that the value of the index did not change much over the study period, suggesting that the degree of socioeconomic inequality remained significant despite great reduction in the maternal mortality ratio.

### Determinants of MMR

Table 2 reports fixed-effect estimates on marginal effects of provincial characteristics on maternal mortality ratio. We controlled for variables of different dimensions step by step in columns (1)–(3) and included them all in the regression in column (4).

**Table 2:** Regression coefficients for maternal mortality ratio

	(1)	(2)	(3)	(4)
Socioeconomic condition				
Average annual household consumption	−0.000			−0.000
	(0.000)			(0.000)
Proportion of illiteracy	0.186			0.887***
	(0.882)			(0.261)
Proportion of college educated	−0.344			− 0.249
	(0.288)			(0.250)
Health resource allocation				
Public budget in health sector per capita		−0.002		− 0.009
		(0.017)		(0.016)
Density of health providers in specialized maternal and child health hospitals		0.055		−4.515
		(6.862)		(4.315)
Density of specialized maternal and child health hospitals		7.495		7.533
		(5.250)		(4.913)
Density of bedsize		−0.076		−0.026
		(0.058)		(0.044)
Access to health care				
Maternity health insurance coverage			0.106	0.090
			(0.095)	(0.070)
Premarital check-up			0.032	0.043
			(0.037)	(0.040)
Maternal health profiles creation rate			0.668	0.619
			(0.432)	(0.388)
Coverage of maternal systematic management			0.025	0.025
			(0.067)	(0.075)
Prenatal check-up			−0.314**	−0.342*
			(0.140)	(0.168)
Postpartum visit rate			−0.358	−0.296
			(0.358)	(0.405)
Hospital delivery rate			−0.682***	−0.778***
			(0.239)	(0.224)
Delivery attended by professionals rate			−1.815**	−1.713**
			(0.687)	(0.646)
Observations	403	403	403	403

Notes: Each column presents a separate regression. The outcome variable is maternal mortality ratio. All specifications control for province fixed effects, year fixed effects, and province-specific year trends. Standard errors are clustered at province level. ***significant at the 1% level; **significant at the 5% level; *significant at the 10% level

Except for the proportion of illiteracy and density of healthcare providers in maternal and child health hospitals, results are largely similar in terms of size and significance across different specifications. When the full set of variables are controlled for, the coefficient of illiteracy proportion increases and becomes significant. That is probably because provinces with low educational attainment were more likely to be poor areas with insufficient maternal health professionals. A one-percentage-point increase in illiteracy proportion was associated with an increase in MMR by 0.887 (*p* < 0.01). We find no evidence that variables regarding health resource allocation exerted an impact on the level of MMR. In contrast, our results show that prenatal check-up rate (*p* = 0.05), hospital delivery rate (*p* < 0.01), and rate of delivery attended by professionals (*p* = 0.02) were negatively associated with the MMR level. A one-percentage-point increase in the aforementioned rates led to a decrease in MMR by 0.342, 0.778, and 1.713, respectively.

### Causes of MMR inequality

Results from the direct decomposition analysis (columns (1)–(3), Table 3) show that marginal effects of maternal health profile creation rate (*p* < 0.01) on the individual component was significantly positive. The effect of average annual household consumption was positive and marginally significant (*p* = 0.06). In contrast, the effect of illiteracy proportion was negative at the margin (*p* = 0.08). Following the interpretation by Kessels and Erreygers [17], this means that higher income level and better healthcare services were associated with a pro-poor change of the inequality of MMR. And higher illiteracy proportion was associated with a pro-rich change of the concentration index. We do not find evidence that other variables were associated with the overall performance of a province with respect to maternal mortality ratio and GDP per capita. Although the effects of hospital delivery rate and rate of delivery attended by professionals were significant at the 5% level in the MMR regressions, their impacts on the inequality index were negligible.

**Table 3:** Decomposition of socioeconomic related inequality in maternal mortality ratio

	Direct decomposition			WDW decomposition
	$\hat{\gamma}$	Logworth		Contribution %
	(1)	(2)	(3)	(4)
Socioeconomic condition			1.078	
Average annual household consumption	0.000*	1.233		5.072
	(0.000)			(13.389)
Proportion of illiteracy	−0.011*	1.103		13.587*
	(0.006)			(4.578)
Proportion of college educated	0.003	0.119		6.198
	(0.009)			(5.750)
Health resource allocation			0.286	
Public budget in health sector per capita	−0.001	0.826		11.993
	(0.001)			(22.169)
Density of health providers	−0.205	0.764		5.447
	(0.146)			(6.434)
Density of maternal and child health hospitals	0.209	0.587		29.597
	(0.181)			(26.293)
Density of bedsize	−0.002	0.512		0.127
	(0.002)			(2.468)
Access to health care			4.483	
Maternity health insurance coverage	0.001	0.095		−3.811
	(0.003)			(3.515)
Premarital check-up	−0.001	0.252		−3.337
	(0.001)			(2.949)
Maternal health profiles creation rate	0.014***	4.260		−14.317
	(0.003)			(10.742)
Coverage of maternal systematic management	0.002	0.137		−1.067
	(0.005)			(4.631)
Prenatal check-up	−0.017	0.926		6.253
	(0.011)			(4.244)
Postpartum visit rate	−0.010	0.632		7.300
	(0.008)			(9.972)
Hospital delivery rate	−0.006	0.334		29.217***
	(0.008)			(11.455)
Delivery attended by professionals rate	−0.008	0.151		15.152*
	(0.021)			(7.661)

Notes: Columns (1)–(3) presents results from the direct decomposition method. The outcome variable is the individual component of the inequality index. Coefficients and standard errors clustered at province level are reported in column (1). Column (2) displays logworth values for each explanatory, and column (3) reports the logworth values for each group of variables of a given dimension. Column (4) presents results from the WDW decomposition method. Contribution percentage and bootstrap standard errors are reported. ***significant at the 1% level; **significant at the 5% level; *significant at the 10% level

The logworth values from the direct regression also point toward maternal profiles creation rate as the important explanatory variable. In general, access to healthcare was the primary cause of socioeconomic inequality in MMR, followed by socioeconomic condition. The importance of health resource allocation came last.

The WDW decomposition analysis (column (4), Table 3) reveals that the degree of inequality in hospital delivery rate contributed 29.22% (*p* < 0.01) to overall inequality in MMR, followed by rate of delivery attended by professionals (15.15%, *p* = 0.06), and illiteracy proportion (*p* = 0.06). Although results of the two decomposition methods are not directly comparable, as they are different in measurement units and response variables of the regressions, the WDW decomposition results also show that access to healthcare was the main cause of MMR inequality, followed by socioeconomic condition.

## Discussion

This study examines the causes of inequality in the maternal mortality ratio at the provincial level in China. Using a longitudinal sample of all provinces in mainland China over an extended time period from 2004 to 2016, we show that socioeconomic related inequality in the MMR has been substantial according to the concentration index. Poor provinces of a lower level of development have persistently suffered more adverse maternal health events than richer provinces.

We explored determinants of the MMR by investigating variation in characteristics regarding socioeconomic condition, health resource allocation, and access to healthcare within provinces over years. We find that the illiteracy proportion was negatively associated with the MMR. In contrast, the hospital delivery rate and rate of delivery attended by a professional were positively associated with the MMR. We then used both the newly proposed direct decomposition method and the conventional WDW approach to explore the causes of MMR inequality. Both of the approaches suggest that access to healthcare was the most important factor in explaining the inequality at the province level, followed by socioeconomic condition. By contrast, we do not find that health resource allocation was an important contributing factor.

The two decomposition methods point to different specific variables as the key cause of inequality, despite the consensus on the importance of access to healthcare. Since the WDW approach restrictively assumes that the relative rank in terms of GDP per capita for a province remain the same in response to changes in explanatory factors, we consider the results from the direct decomposition method more reliable. We find that the rate of maternal health profile creation was positively (*p* < 0.01) associated with the joint performance of a province in terms of the MMR and GDP per capita. This finding suggests that an equal increase in the average rate of maternal health profile creation can help address the persistent MMR inequality by reducing the ratio more among poor provinces.

This study contributes to the literature on determinants of health inequality by providing an analysis at the provincial level in China [18, 25–27]. We note that the importance of the factors to health inequalities at the aggregate-level could be different from that at the individual-level. Results from individual level analyses consistently emphasized the important contribution of socioeconomic conditions, such as, household income, educational attainment, and rural residence, to the overall health inequality [18, 26–28]. Although our results also recognize the role of socioeconomic status, we observe a much weaker protective effect of these variables and a stronger effect from access to healthcare on the MMR inequality in China. One important exception among individual level analyses is Lin [25], which examined the causes of narrowed infant health disparity in the US at the individual level and concluded that increases in access to medical care was the most important factor in explaining the closing gap.

By contrast, our findings are more consistent with studies focusing on health differences at aggregate levels. Randive et al. [29] examined inequality in maternal mortality at the district level in the context of India and found that male illiteracy and access to healthcare were the two most important contributors. Using data from Indonesia, Cameron et al. [30] posits that socio-economic status was not a significant contributing factor for differences in maternal mortality across provinces. However, health resource allocation and access to healthcare were the most important contributors.

### Policy implications

From a policy perspective, our results suggest that the continuity of maternal health management rather than just delivery services may benefit pregnant women in rural areas more, which could be the focus of future policy interventions. In the direct decomposition analysis, the maternal health profile creation rate is significantly positive with the largest logworth value. Setting up a maternal health profile for the pregnant is considered as the starting point of providing continued care. In China, pregnant women are encouraged to decide their production hospitals and to set up their maternal health profiles at their chosen hospitals at their early pregnancy. Such profiles record tests results, help remind regular prenatal care checkups, and thus enable systematic and comprehensive management of pregnancy and fetal development.

Moreover, disparities in the quality of care may also be responsible for the observed long-lasting socioeconomic related MMR inequality. Substantial government inputs through rounds of programs and policies have led to rapid increases in hospital delivery rates from an average of 81.68% in 2004 to 99.47% in 2016. However, delivery service utilization at hospitals did not help eliminate socioeconomic related MMR inequality. Studies have shown substantial gaps in healthcare quality for treatments of common clinical conditions across tertiary hospitals in China [31, 32]. Low quality of maternity and obstetric care in less developed areas might have also prevented investments in service utilization, such as hospital delivery, to exert a strong impact on health outcomes.

### Limitations

Our study is subject to some limitations. First, due to lack of data availability, we do not have access to some other factors that are also extensively discussed in the literature, such as cultural factors, and the quality of healthcare; although rural residents and other urban residents may also be able to receive some reimbursements for maternal and child health services to different extent in recent years, we were not able to capture information on their access to such benefits using merely the variable maternity health insurance coverage. Second, we note that our results were produced using aggregate level analysis, which cannot be directly inferred as causality or inferences at individual level.

## Conclusions

This paper adds to the health equity literature by examining the concentration index of the MMR at the province level and decomposing the inequality index using an approach that recognizes its “two dimensional” feature. We find that access to healthcare was the most important factor in explaining the persistent MMR inequality in China, followed by socioeconomic condition. We do not find evidence that health resource allocation was a contributing factor. Our results point out that increasing access to health care and reducing the illiteracy proportion can both help address the persistent MMR inequality. More cost-effectiveness evidence is needed to support the choice of relevant interventions and programs, which could be a direction of future research.

## Supplementary information

**Additional file 1: Table S1.** Definition of variables used in the analysis.

## Data Availability

The datasets generated and/ or analyzed during the current study are available in the public domain from various issues of the China Health Statistics Yearbook, China Statistics Yearbook and China Population, and Employment Statistics Yearbook.

## Abbreviations

MMR: Maternal mortality ratio
SDGs: Sustainable Development Goals
CI: Concentration index
GDP: Gross domestic product
