# Supplementary material for: Anatomy of provincial level inequality in maternal mortality in China during 2004–2016: a new decomposition analysis
Source: BMC Public Health. 2020 May 24;20:758. doi: 10.1186/s12889-020-08830-2 (PMC7245773; doi:10.1186/s12889-020-08830-2)
Supplement: Supplementary file 1 — Additional file 1: Table S1. Definition of variables used in the analysis. [file 12889_2020_8830_MOESM1_ESM.docx]

**Supplementary materials**

Table S1. Definition of variables used in the analysis

| Variables | Definition |
| --- | --- |
| Maternal mortality ratio  (MMR) | The number of maternal deaths per 100,000 live births |
| Gross domestic product  (GDP) per capita | The final products by all residents divided by resident population |
| Average annual household  consumption | The average total annual household expenditures on final consumption of goods and services |
| Proportion of illiteracy | The proportion of people with no years of schooling among those aged above 6. |
| Proportion of college educated | The proportion of people with college equivalent degrees among those aged above 6. |
| Public budget in health sector per capita | The funds which the government has raised and distributed to health sector divided by resident population |
| Density of health providers  in specialized maternal and child health hospitals | The total number of health professionals (including physicians, nurses, and technicians) in specialized maternal and child health hospitals per 10,000 population |
| Density of specialized  maternal and child health hospitals | The total number of maternal and child health hospitals per 1,000,000 population |
| Bed size in maternal and child health hospitals per 100,000 population | The total bed sizes of maternal and child health hospitals per 100,000 population |
| Maternity health insurance coverage | The proportion of urban employed population under social health insurance who has opted in the maternity health insurance |
| Premarital check-up rate | The proportion of people undertaking premarital check-up among those registered marriage. |
| Maternal health profiles creation rate | The number of health profiles created by maternal care providers over live births |
| Coverage of maternal systematic management | The number of women over live births, who received early pregnancy test, at least five prenatal check-ups, delivery attended by professionals and postpartum visits up to 42 days after giving birth |
| Prenatal check-up rate | The number of women over live births, who received at least one prenatal check-up. |
| Postpartum visit rate | The number of women over live births, who received at least one postpartum visit. |
| Hospital delivery rate | The number of live births delivered at hospitals with midwifery qualification, over total live births |
| Delivery attended by professionals rate | The total number of deliveries attended by skilled staff over live births |
